# A tale of two parts of Switzerland: regional differences in the impact of the COVID-19 pandemic on parents

**DOI:** 10.1186/s12889-021-11315-5

**Published:** 2021-06-30

**Authors:** Michelle Seiler, Georg Staubli, Julia Hoeffe, Gianluca Gualco, Sergio Manzano, Ran D. Goldman

**Affiliations:** 1 Pediatric Emergency Department, University Children’s Hospital Zurich, Steinwiesstrasse 75, 8032 Zurich, Switzerland; 2 Pediatric Emergency Department, Inselspital University Hospital of Bern, Bern, Switzerland; 3 Pediatric Emergency Department, Pediatric Institute of Italian part of Switzerland, Bellinzona, Switzerland; 4 Pediatric Emergency Department, Geneva University Hospitals and Faculty of Medicine, University of Geneva, Geneva, Switzerland; 5 The Pediatric Research in Emergency Therapeutics (PRETx) Program, Division of Emergency Medicine, Department of Pediatrics, University of British Columbia, and BC Children’s Hospital Research Institute, Vancouver, BC Canada

**Keywords:** COVID-19, SARS-CoV-2, Parental concern, Switzerland, Emergency department

## Abstract

**Background:**

We aimed to document the impact of the coronavirus disease 2019 (COVID-19) pandemic on regions within a European country.

**Methods:**

Parents arriving at two pediatric emergency departments (EDs) in North of Switzerland and two in South of Switzerland completed an online survey during the first peak of the pandemic (April–June 2020). They were asked to rate their concern about their children or themselves having COVID-19.

**Results:**

A total of 662 respondents completed the survey. Parents in the South were significantly more exposed to someone tested positive for COVID-19 than in the North (13.9 and 4.7%, respectively; *P* <  0.001). Parents in the South were much more concerned than in the North that they (mean 4.61 and 3.32, respectively; *P* <  0.001) or their child (mean 4.79 and 3.17, respectively; *P* <  0.001) had COVID-19. Parents reported their children wore facemasks significantly more often in the South than in the North (71.5 and 23.5%, respectively; *P* <  0.001).

**Conclusion:**

The COVID-19 pandemic resulted in significant regional differences among families arriving at EDs in Switzerland. Public health agencies should consider regional strategies, rather than country-wide guidelines, in future pandemics and for vaccination against COVID-19 for children.

**Supplementary Information:**

The online version contains supplementary material available at 10.1186/s12889-021-11315-5.

## Background

**Fig. 1:**
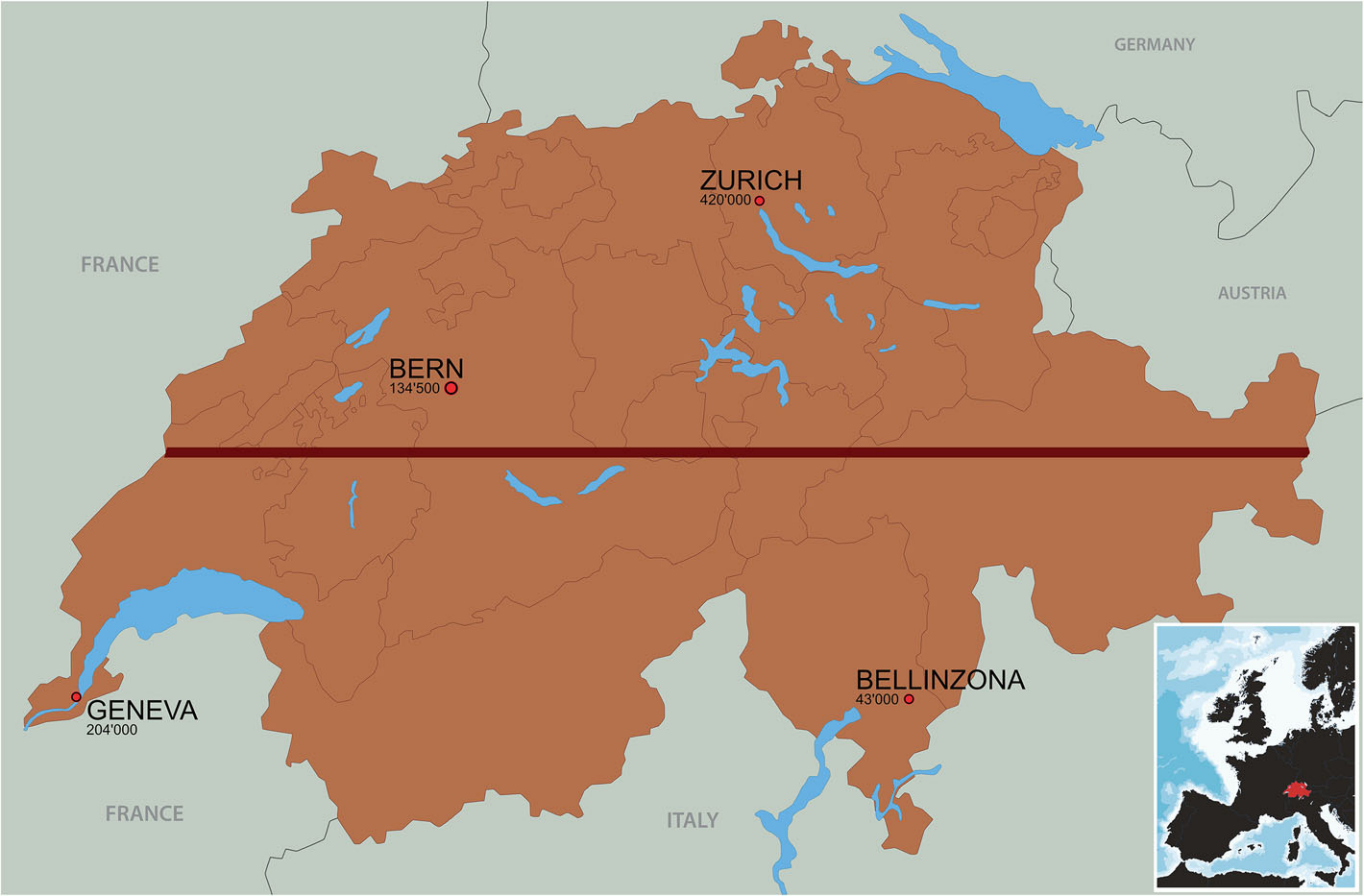
A map of Switzerland and surrounding countries including population sizes. The red line divides the country into North (including Zurich and Bern) and South (including Bellinzona and Geneva). (Software affinity photo, version 1.8.4, https://affinity.serif.com/de/photo/)

The first cases of severe acute respiratory syndrome coronavirus 2 (SARS-CoV-2) in Europe were recorded on January 24, 2020 [1] with a subsequent rapid rise in the number of confirmed cases with significant morbidity and mortality [2]. Italy and France, neighbors of Switzerland, were overwhelmed by the coronavirus disease 2019 (COVID-19) as of February 2020 [3, 4]. The capital of Switzerland is Bern in central Switzerland, the largest city is Zurich in northern Switzerland, and the second-largest city is Geneva in the West. The second-largest city in the canton of Ticino in the South of Switzerland is Bellinzona (Fig. 1).

The level of concern regarding COVID-19 in Switzerland varied between regions. The first COVID-19 case in Switzerland was in the canton of Ticino, bordering Italy, resulting in the canton imposing noteworthy public health measures, including closing the border with Italy. On March 17, 2020, the Swiss government declared a national lockdown with closing of schools and a shutdown of public life, which partially ended in the beginning of May 2020.

The South and West of Switzerland documented higher morbidity and mortality rates from COVID-19 compared to Zurich and Bern [5–7]. By the end of the lockdown, Ticino and Geneva had each recorded three times more deaths than Zurich and Bern. Geneva counted 1200 hospitalized patients, Ticino 700, and Zurich and Bern tallied 300 patients each [6].

The COVID-19 pandemic resulted in a decrease in pediatric emergency medicine attendances from 30% up to 76%, mainly for communicable infections [8, 9].

The objective of this study was to evaluate the differing impact of COVID-19 on parents’ attitudes when attending an emergency department (ED) during and after the lockdown in different regions in Switzerland. Our hypothesis was that regional differences exist and that parents in cities neighboring Italy (Bellinzona) and France (Geneva) would be more concerned about their children or themselves having COVID-19 than parents in the North of Switzerland (Zurich, Bern).

## Methods

This investigation is part of the COVID-19 International Parental Attitude Study (COVIPAS) Group for parents (including all caregivers) presenting their child to one of 17 EDs in six countries [10], including the main regional hospitals in four regions in Switzerland: Bern, Geneva, Zurich, and Bellinzona. Parents were approached to participate in this survey by healthcare team members, as well as posters that were placed in waiting areas and patient rooms. Parents used their personal smartphones to fill out the questionnaire by logging into a secure online platform based on REDCap metadata-driven software (Vanderbilt University). A waiver of consent was provided by the Swiss ethics committee because responding to the survey was considered consent to participate and because no identifiers were collected. Languages available to complete the survey questionnaire were German, French, Italian, and English.

Sites joined recruitment in a staggered fashion between April 10 and June 30, 2020. Bern started April 10, Geneva April 17, Zurich April 18, and Bellinzona May 22. Only one parent completed the survey because only one parent was allowed to accompany the child due to restrictions on family visitation in EDs.

The study-specific questionnaire was developed to include questions on attitudes to COVID-19 (see Supplementary file 1). The survey was designed to assess parental opinions during the pandemic. The survey was tested for clarity a priori by 20 individuals. Feedback led to revisions and development of the final survey.

Demographic questions included the child’s age, whether a family member was exposed to someone who had tested positive for SARS-CoV-2 and whether the child wore a facemask before coming to the ED.

We asked parents to answer five questions about the impact of COVID-19 on their family. Five questions focused on issues about the impact of COVID-19 with a Likert scale (0: not at all; 10: the most I have ever been): (1) “How worried are you that your child has Coronavirus (COVID-19)?” (2) “How worried are you that you have Coronavirus (COVID-19)?” (3) “How worried are you about missing work?” (4) “How worried are you about your child missing school?” (5) “Has Coronavirus (COVID-19) led you to lose income due to loss of job or inability to work?”

### Statistical analysis

Statistical analyses were performed with IBM® SPSS® statistics version 24 (IBM, Armonk, NY). Basic descriptive statistics and frequencies were used to describe all variables and compare the North of Switzerland (Zurich and Bern) to the South (Bellinzona and Geneva reflecting proximity to Italy and France). The independent t-test was used for comparing normally distributed continuous variables, and Chi-square or Fisher’s exact test for categorical variables. For all tests, *P*-values of less than 0.05 were considered statistically significant.

## Results

A total of 662 respondents participated in the study and completed the survey questionnaire online. Certain questions were not answered by all participants, accounting for nine (1.4%) unknown responses to demographic questions and 48 (7.3%) regarding the extent of concern. The mean age of children was 7.4 (Standard Deviation (SD) = 4.5) years; children in the South were older than children in the North (8.2 years and 7.1 years, *P* = 0.011). A comparison between families from the North and those from the South are provided in Table 1. No significant differences were found in the proportions of children’s sex, children having a chronic illness, use of long-term medications, or who completed the survey. In the South, more respondents completed the surveys after the lockdown than in the North (85.8% compared to 54.6%, *P* <  0.001).

**Table 1:** Demographic information and number of respondents in North and South Switzerland. SD = standard deviation

	Number of respondents	North	South	*p* value
Child’s mean age in years (SD)	659	7.1 (±4.5)	8.2 (±4.4)	*0.011*
Child’s sex Male	660	288/518 (55.6%)	68/142 (47.9%)	0.102
Child has a chronic illness	659	35/518 (6.8%)	8/141 (5.7%)	0.644
Child uses long-term medication	660	54/519 (10.4%)	14/141 (9.9%)	0.869
Survey was completed by the Mother	659	336/518 (64.9%)	99/141 (70.2%)	0.235
Family member was exposed to COVID-19	653	24/516 (4.7%)	19/137 (13.9%)	*< 0.001*
Survey completed out after lockdown (May 11 – June 30, 2020)	662	284/520 (54.6%)	121/142 (85.8%)	*< 0.001*

Significant *p*-values are emphasized using italics

Parents in the South reported an exposure to someone who tested positive for COVID-19 significantly more often than in the North (13.9 and 4.7%, respectively; *P* <  0.001). Parents whose families had been exposed to COVID-19 were significantly more worried that they had COVID-19 themselves (Table 2).

**Table 2:** Comparing families reporting exposure to a confirmed case of COVID-19, and families who report no exposure to a confirmed case of COVID-19 before arriving at the Emergency Department. Subset of families that reported concern about having COVID-19 (Likert scale 1–10, excluding families who were not worried). Also analyzing subsets of families with concerns regarding work, loss of income, and their child missing school. SD = standard deviation

	Reported Exposure to Someone with Confirmed COVID-19	Not known Exposure to Someone with Confirmed COVID-19	*p* value
Parents concerned their children have COVID-19 (Likert scale 1–10), mean (SD)	4.41 (±2.87)	3.51 (±2.41)	0.057
Number of parents very worried their children have COVID-19 (Likert scale 7–10), n (%)	7/43 (16.3%)	41/584 (7%)	*0.038*
Parents concerned they have COVID-19 (Likert scale 1–10), mean (SD)	5.04 (±2.54)	3.56 (±2.35)	*0.002*
Parents very concerned they have COVID-19 (Likert scale 7–10), n (%)	8/43 (18.6%)	45/583 (7.7%)	*0.022*
Parents concerned about missing work (Likert scale 1–10) (SD)	4.77 (±3.06)	4.3 (±2.8)	0.413
Parents concerned about their children missing school (Likert scale 1–10) (SD)	4.71 (±3.07)	4.73 (±2.83)	0.970
Parent report loss of income due to COVID-19, n (%)	11/43 (25.6%)	194/599 (32.4%)	0.355

Significant *p*-values are emphasized using italics

The level of concern about COVID-19 was significantly higher in the South than the North about both parents and their children having COVID-19 (Table 3). Parents from the South whose families had been exposed to COVID-19 were mostly concerned that they or their children had COVID-19. Forty-three percent of parents stated that they were not concerned at all about their children having COVID-19; those parents were mainly from the North (North 46.5%, South 31.3%, *P* = 0.002). In the North, 56.9% of parents were not worried they had COVID-19 compared to 37.8% in the South (*P* <  0.001).

**Table 3:** The impact of COVID-19 reported by families in the North and the South of Switzerland, including families who reported concern about having COVID-19 (Likert scale 1–10, excluding families who were not concerned). SD = standard deviation

	North	South	*p* value
Parents concerned their children have COVID-19 (Likert scale 1–10), mean (SD) Family exposed to COVID-19: Parents concerned their children have COVID-19 (Likert scale 1–10), mean (SD)	3.17 (±2.23) 3.31 (±2.43)	4.79 (±2.68) 5.31 (±2.96)	*< 0.001*
Parents very concerned their children have COVID-19 (Likert scale 7–10), n (%) (*n* = 633) Family exposed to COVID-19 (*n* = 627)	22/495 (4.4%) 2/24 (8.3%)	26/132 (19.7%) 5/19 (26.3%)	*< 0.001*
Parents concerned they have COVID-19 (Likert scale 1–10), mean (SD) Family exposed to COVID-19	3.32 (±2.16) 4.45 (±2.58)	4.61 (±2.71) 5.47 (±2.5)	*< 0.001*
Parents very worried they have COVID-19 (Likert scale 7–10) Family exposed to COVID-19	27/493 (5.5%) 2/27 (7.4%)	26/133 (19.5%) 6/26 (23.1%)	*< 0.001*
Parents concerned about missing work (Likert scale 1–10), mean (SD) Family exposed to COVID-19	4.02 (±2.74) 4.44 (±3.09)	5.5 (±2.87) 5.5 (±3.07)	*< 0.001*
Parents concerned about their child missing school (Likert scale 1–10), mean (SD) Family exposed to COVID-19	4.47 (±2.81) 3.86 (±3.09)	5.48 (±2.82) 5.90 (±2.77)	*0.006*
Parents report child wearing facemask when coming to the Emergency Department Family exposed to COVID-19	121/515 (23.5%) 3/24 (12.5%)	98/137 (71.5%) 13/19 (68.4%)	*< 0.001*
Parent report lost income due to COVID-19 Family exposed to COVID-19	160/506 (31.6%) 8/24 (33.3%)	45/136 (33.1%) 3/19 (15.8%)	0.760

Significant *p*-values are emphasized using italics

The proportions of children reported as wearing a facemask when coming to the ED were similar between those with families exposed to confirmed cases of COVID-19 and those not reporting exposure (37.2 and 33%, respectively, *P* = 0.603). Children wore facemasks significantly more often in the South than in the North (71.5 and 23.5%, respectively; *P* <  0.001; Table 3).

A third (31.6%) of all parents stated that they had lost income because of COVID-19, with similar proportions in the North and South of Switzerland. However, 49.2% in the North and 47% in the South were not concerned about work (*P* = 0.695). Of those who were concerned, the concern level of parents about missing work was higher in the South, independent of whether the family had been exposed to COVID-19 or not (mean 5.5 and 4.02, respectively; *P* <  0.001). However, parents who lost income due to COVID-19 were more worried about missing work than those who did not lose income (mean 2.65 and 2.03, respectively; *P* = 0.015). Half of the families in this study were not concerned that their child missed school (North 53.5%, South 39.6%, *P* = 0.005), and the level of parental concern in the South was higher than in the North (mean 5.48 and 4.47, respectively; *P* = 0.006; Tables 2 and 3).

## Discussion

We report that within Switzerland, one of the smallest countries in Europe, significant regional differences existed in the impact of COVID-19 on parents’ attitudes as well as actions. Those living in cities bordering countries most heavily hit by COVID-19, Italy and France, were more affected than parents in the North of Switzerland. We found that parents in the South had significantly more exposure to someone who had tested positive for COVID-19, were significantly more worried that they or their children had COVID-19 when arriving at an ED, were worried about missing work and their children missing school. This may have resulted in wearing facemasks on their children so early in the first peak of the pandemic and significantly more often than did parents of children from the North.

Higher morbidity and mortality rates from COVID-19 were documented in the cantons of Ticino and Geneva compared to Zurich and Bern [5–7]. Swiss authorities imposed national restrictions and we stipulate that the differences we report cannot be explained by different restrictions in these regions. The broader effect on parents’ reports in the South is likely a result of the higher risk of a family member being exposed to someone who tested positive for COVID-19 (three times higher than in the North).

In the North, almost half of parents were not concerned that their child had COVID-19, possibly reflecting Swiss authorities’ effort to diminish public apprehension about the illness. At the time of the survey, public health messaging suggested the illness was mainly unsafe for the elderly and for adults with chronic conditions rather than children. Additionally, authorities initially suggested that wearing facemasks was not necessary and that social distancing and frequent hand washing were more important measures [11]. An online survey of residents in Switzerland during lockdown period reported that most people in the North and East of Switzerland approved authorities’ actions: only 11% reported wearing facemasks, compared to 24% in the West and 48% in the South [12]. In our survey, 70% of parents in the South responded that their children wore a facemask when arriving at the ED, suggesting heightened concern about them or their children already having COVID-19 or apprehensions about contracting the infection in the hospital. This finding supports the notion that those who were more worried about COVID-19 were also more engaged in precautionary measures, like using facemasks [13].

Switzerland is known for residents’ high satisfaction with quality of life in all regions of the country [14] and concerns about unemployment, expressed more by men than by women, vary regionally [15, 16]. The pandemic resulted in economic distress with noticeable uptick in unemployment [17, 18]. One third of all parents in our study stated that they lost income, and they were more concerned about missing work, compared to families maintaining their income. In the South, concerns about missing work were comparable with those about missing school (mean 5.5, mean 5.48), yet in the North parents were more concerned about their children missing school (mean 4.5) than they were concerned about missing work (mean 4; *P* = 0.044).

Our findings are consistent with reports from other countries. In one report from the USA, almost a third of parents were worried that school closure affected their children’s mental and emotional health [19]. In an online survey with 2200 participants in the United Arab Emirates, 35% were worried about contracting COVID-19, and even higher concern for grandparents (75%) and for children (65%) [20]. As an anticipated social norm, parents consider their children’s health as a priority over their family’s economic stability [19, 20].

Our study has a number of limitations. First, the parents who completed the survey do not represent all parents at the sites where the study was conducted. Secondly, a smartphone was required to complete the survey, which may have excluded a small percentage of parents from responding. Thirdly, sites began recruitment in a staggered fashion, and in Bellinzona, recruitment only began after the end of the Swiss lockdown, which may have led to an underestimation of the level of concern in this region. Also, the number of respondents was higher in the North than in the South, which might be due to larger EDs or could be a sign that parents in the North were more willing to take time to fill out a survey during their stay at the ED.

## Conclusions

In conclusion, the impact of COVID-19 on parents’ attitudes reflects significant regional differences within Switzerland. In the South, hard hit by the pandemic, parents were more often exposed to someone who tested positive for COVID-19, were significantly more concerned that they or their children had COVID-19, and were more likely to adopt protection measures. Intercountry regional evaluation of families’ considerations and actions is important, governments should consider recommendations for specific regions, rather than imposing public health broadly on the entire country. These lessons should guide the developing strategies for future dissemination of vaccination against COVID-19 in children, as well as future pandemics.

## Supplementary Information

**Additional file 1: Supplementary file 1.** Questionnaire used for this study (English version).

## Data Availability

The datasets analysed during the current study are available from the corresponding author on reasonable request.

## Abbreviations

COVID-19: coronavirus disease 2019
ED: emergency department
SARS-CoV-2: severe acute respiratory syndrome
SD: standard deviation
