# Supplementary material for: A tale of two parts of Switzerland: regional differences in the impact of the COVID-19 pandemic on parents
Source: BMC Public Health. 2021 Jun 30;21:1275. doi: 10.1186/s12889-021-11315-5 (PMC8242280; doi:10.1186/s12889-021-11315-5)
Supplement: Supplementary file 1 — Additional file 1: Supplementary file 1. Questionnaire used for this study (English version). [file 12889_2021_11315_MOESM1_ESM.docx]

| **The results of this survey will be collected anonymously**  This survey is offered to everyone coming to our Emergency Department, and is not related to your child’s symptoms or condition  Your answers will not change your child’s care  This study takes place in several hospitals and clinics. Data will be shared with other scientists without any identifying information. |
| --- |
| **DEMOGRAPHICS**  Your child’s age: years _______ months _______ |
| Your child’s gender M / F / X |
| What is your relationship to the patient?  mother,  father,  sibling,  other ____________________ |
| Does your child have a chronic illness? Y / N  If yes, list ________________________________ |
| Does your child take regular prescription medications (e.g., pills, inhalers, injections)? Y / N |
| Was anyone at home exposed to someone WHO TESTED POSITIVE for Coronavirus (COVID-19)? Y/N  IF YES : Who is ? ___________________  Please note relation to your child (e.g. "sister"). Do NOT put their name |
| **EMERGENCY VISIT**  What is the main reason you brought your child to the emergency department today ? ___________ |
| **PROTECTION**  Which of the following did your child use today before coming to the emergency department? [Choose all applicable]  Surgical face mask  Filtered face mask (called N95)  Other face mask  Other ________________________________________ |
| **Between 0-10** (0- not at all 10 – The most I have ever been)  How worried are you that your child has Coronavirus (COVID-19) ? __________  How worried are you that you have Coronavirus (COVID-19)? __________  How worried are you that your child has INFLUENZA (the FLU) ? __________  How worried are you that you have INFLUENZA (the FLU)? __________  How worried are you about missing work ________  How worried are you about your child missing school _______ |
| Has Coronavirus (COVID-19)led you to lose income due to loss of job or inability to work? Y/N |
| Please share any other concerns related to Coronavirus (COVID-19) that you may have:  _______________________________ |
| Thank you for participating. |
